# System-Level Barriers to National Institute for Health and Care Excellence (NICE) Guideline Compliance in Distal Radius Fracture Management: A Retrospective Audit Study

**DOI:** 10.7759/cureus.97621

**Published:** 2025-11-24

**Authors:** Mahmoud Mersal, Mohamed Elmarakby, Mohamed Elgendy, Sara Elsaidy, Osama Embaby, Ahmed Elmahdi, Tamer Sweed

**Affiliations:** 1 Orthopaedics and Trauma, University Hospitals Birmingham (UHB) NHS Foundation Trust, Birmingham, GBR; 2 Orthopaedics, University Hospitals Birmingham (UHB) NHS Foundation Trust, Birmingham, GBR; 3 Orthopaedics, Sandwell and West Birmingham Hospitals NHS Trust, Birmingham, GBR; 4 Orthopaedics and Trauma, Salford Royal NHS Foundation Trust, Manchester, GBR

**Keywords:** distal radius fracture, nice guidelines, orthopaedic audit, quality improvement, surgical timing, trauma surgery

## Abstract

Background

Distal radius fractures are among the most common orthopaedic injuries, particularly in the elderly population. The UK's National Institute for Health and Care Excellence (NICE) provides clear management guidelines, yet adherence remains inconsistent. This quality improvement project evaluated and enhanced compliance with NICE standards for the management of dorsally angulated distal radius fractures at University Hospitals Birmingham NHS Foundation Trust, a major trauma centre in Birmingham, United Kingdom.

Methods

A two-cycle audit was undertaken using the Plan-Do-Study-Act (PDSA) framework. Cycle 1 (May-August 2020) retrospectively reviewed 30 adults to assess compliance with NICE recommendations for manipulation. Following an educational intervention, Cycle 2 (June-August 2023) evaluated 43 adults requiring surgery, focusing on compliance with NICE surgical timing standards. Data were obtained from electronic patient records.

Results

In Cycle 1, 90% (27/30) of patients received manipulation in the emergency department (ED), indicating good baseline compliance. In Cycle 2, only 11.6% (5/43) underwent surgery within the NICE-recommended timeframe - 10% (4/40) for intra-articular fractures (target ≤ 72 hours) and 33.3% (1/3) for extra-articular fractures (target ≤ 7 days). The mean time to surgery was 8.6 days for intra-articular and 6.3 days for extra-articular fractures. Limited theatre capacity accounted for 73.7% (28/38) of delays.

Conclusions

Educational measures improved awareness but did not resolve system-level barriers. Theatre capacity and list allocation remain the main obstacles to timely surgical management. Achieving full compliance with NICE guidance requires combining educational reinforcement with structural and resource-based solutions.

## Introduction

Distal radius fractures are among the most frequently encountered injuries in orthopaedic practice, accounting for a significant proportion of fractures treated in emergency departments (EDs) worldwide [1]. These injuries, particularly common among older adults, can lead to substantial pain, loss of function, and reduced quality of life if not managed appropriately [2]. The incidence in the United States is estimated at approximately 640,000 cases annually, showing a bimodal distribution that includes young males sustaining high-energy trauma and older females with osteoporotic bone [1]. Recent European data suggest that distal radius fractures represent around 17.5% of all adult fractures, making them the most common fracture type in this population [3].

To standardise care and optimise outcomes, the National Institute for Health and Care Excellence (NICE) has published evidence-based guidance for the management of fractures [4]. For dorsally displaced distal radius fractures in skeletally mature adults, NICE recommends initial manipulation and plaster casting as the first-line definitive treatment when acceptable alignment can be achieved [4]. This conservative approach aims to restore anatomical configuration and avoid unnecessary surgical intervention.

For patients requiring surgical fixation, NICE specifies time-sensitive standards to minimise complications and enhance recovery. Surgery should be performed within 72 hours for intra-articular fractures and within 7 days for extra-articular fractures. When fixation is necessary, K-wire fixation is recommended if closed reduction is achievable and the articular surface remains intact, while open reduction and internal fixation (ORIF) should be considered when closed reduction fails [4].

Despite the availability of such guidance, consistent compliance remains a challenge across healthcare systems [5,6]. Gaps between evidence and practice can result in variation in care, suboptimal outcomes, and inefficient resource use [7]. Barriers to implementation are often multifactorial, including lack of awareness, organisational pressures, and limited theatre capacity [8,9]. The Consolidated Framework for Implementation Research (CFIR) highlights the influence of both internal factors (culture, resources) and external factors (policy, regulation) on adherence [10].

This quality improvement project was therefore conducted to evaluate our institution’s compliance with NICE guidelines in the management of dorsally angulated distal radius fractures, implement targeted interventions, and reassess performance. The objectives were to measure baseline compliance with manipulation and surgical timing standards, identify barriers to adherence, and evaluate the effect of educational and organisational interventions through a two-cycle audit process.

## Materials and methods

Study design and setting

This quality improvement project was designed as a retrospective, two-cycle audit structured around the Plan-Do-Study-Act (PDSA) methodology over two distinct cycles [11]. The project was conducted at University Hospitals Birmingham NHS Foundation Trust, a major trauma centre in the United Kingdom serving a diverse urban and suburban population. It was registered with the hospital’s clinical governance team as a service evaluation (Audit Codes: CARMS-16530 and CARMS-20612). In accordance with UK National Health Service (NHS) policy for service evaluations, formal research ethics committee approval was not required, and individual patient consent was waived.

PDSA methodology

The PDSA framework provided a structured approach for iterative learning and continuous improvement through sequential cycles of planning, implementation, evaluation, and refinement [11].

Cycle 1: Baseline Assessment (May-August 2020)

The first cycle, conducted between 1 May 2020 and 31 August 2020, during the COVID-19 pandemic, at a time when national restrictions and NHS-wide prioritisation of urgent and emergency care affected theatre capacity and case scheduling. The first cycle aimed to assess baseline compliance with the NICE guideline recommending manipulation for dorsally displaced distal radius fractures in skeletally mature adults. During the Plan phase, the audit question and standards were defined. The Do phase involved a retrospective review of patient records to collect data on initial management practices. The Study phase analysed the collected data to identify compliance rates and reasons for non-adherence. This revealed that 90% of patients received manipulation as recommended, while 10% proceeded directly to surgery without documented attempts at manipulation.

Act Phase: Educational Intervention (2020-2023)

Based on the findings from Cycle 1, a multifaceted educational intervention was implemented to address the 10% non-compliance rate. The intervention included circulating an email to all Trauma and Orthopaedic registrars through the departmental administrative lead to raise awareness of the NICE guidance, delivering dedicated teaching sessions on evidence-based distal radius fracture management, and developing an internal management pathway to serve as an institutional reference for standardised practice across the department. These initiatives aimed to strengthen awareness, improve documentation, and ensure consistency in decision-making for dorsally displaced distal radius fractures.

Cycle 2: Re-Audit and Surgical Timing Assessment (June-August 2023)

The second cycle, conducted between 3 June 2023 and 30 August 2023, was performed to evaluate the impact of these interventions and to expand the audit focus toward compliance with NICE surgical timing standards. The Plan phase redefined the audit objective to assess adherence to the recommended surgical timeframes - within 72 hours for intra-articular fractures and within seven days for extra-articular fractures. The Do phase comprised retrospective data collection from all patients who underwent operative fixation. The Study phase revealed marked non-compliance with surgical timing standards, identifying significant delays in access to theatre. The subsequent Act phase, therefore, emphasised organisational-level recommendations, specifically targeting theatre capacity, list allocation, and operational scheduling.

Participants and selection criteria

Patients were included if they were adults (skeletally mature, aged ≥16 years) treated for a dorsally displaced distal radius fracture during either audit period. Patients were excluded if they were skeletally immature (<16 years) or had non-dorsally displaced fractures. In Cycle 1, 57 patient records were reviewed, of which 30 met the inclusion criteria. In Cycle 2, 83 records were reviewed, and 43 cases were included.

Data collection and outcomes

Data were extracted retrospectively from the hospital’s electronic patient-record system. For Cycle 1, the primary outcome was the proportion of patients receiving manipulation in the ED as per NICE guidance. Additional variables included patient demographics (age, gender), fracture characteristics (AO/OTA classification, mechanism of injury), anaesthetic technique for manipulation, post-reduction outcomes, and documented reasons or contraindications for omitting manipulation, such as ipsilateral fractures.

For Cycle 2, the primary outcomes were the proportions of patients undergoing surgery within the NICE-recommended timeframes, ≤72 hours for intra-articular fractures and ≤7 days for extra-articular fractures. Additional data collected included demographics, fracture pattern, type of surgical fixation (open reduction and internal fixation (ORIF) versus K-wire fixation), time from injury to surgery, temporal distribution of delays, and recorded reasons for delay.

Statistical analysis

Data were analysed using descriptive statistics. Categorical variables were summarised as frequencies and percentages, while continuous variables were presented as means and ranges. Compliance rates were calculated as the proportion of patients meeting NICE standards within each cycle. As this was a quality improvement project, inferential statistical testing was not undertaken. Instead, performance was compared between audit cycles and benchmarked against the NICE guideline to identify gaps and direct further improvement.

## Results

Cycle 1: baseline assessment (manipulation compliance)

Patient Demographics and Fracture Characteristics

Between 1 May 2020 and 31 August 2020, 57 patient records were reviewed; 30 met the inclusion criteria of skeletally mature adults with dorsally displaced distal radius fractures. The mean age was 64.3 years (range 18-89), and females comprised 73.3% (22/30), consistent with the well-known predominance of these injuries among post-menopausal women [1]. Low-energy falls from standing height accounted for 80% (24/30) of cases, while 20% (6/30) resulted from higher-energy trauma such as road-traffic collisions or falls from height.

According to the AO Foundation/Orthopaedic Trauma Association (AO/OTA) classification, extra-articular (Type A) fractures represented 60% (18/30), partial-articular (Type B) 16.7% (5/30), and complete-articular (Type C) 23.3% (7/30). This reflects the typical distribution encountered in a major trauma centre serving a mixed population.

Compliance With Manipulation Guidelines

Of the 30 included patients, 27 (90%) underwent manipulation and plaster immobilisation in the ED, aligning with NICE guidelines recommendation 1.4.5. Three patients (10%) proceeded directly to the operative pathway without documented manipulation attempts, constituting the main area of non-compliance.

Anaesthetic Techniques for Manipulation

Bier’s block was used in 18 cases (66.7%), haematoma block in seven (25.9%), and procedural sedation in two (7.4%). No patient received nitrous oxide (“gas and air”) alone, which is consistent with NICE 1.3.2 discouraging its solo use.

Outcomes and Analysis of Non-compliance

Acceptable reduction was achieved in 77.8% (21/27) of manipulations; six patients (22.2%) lost reduction and later required surgery. Of the three non-manipulated cases, one (33.3%) had a justified contraindication - an ipsilateral olecranon fracture. The remaining two (6.7% of the total) had no documented reason for bypassing manipulation. Retrospective discussion suggested either a perception of irreducibility or a lack of awareness of NICE guidance. The absence of contemporaneous documentation prevented firm conclusions.

Cycle 2: re-audit (surgical timing compliance)

Patient Demographics and Fracture Characteristics

Between 3 June 2023 and 30 August 2023, 83 records were reviewed; 43 met the inclusion criteria for surgical fixation of dorsally displaced distal radius fractures. Mean age was 66.8 years (range 22-87); females comprised 69.8% (30/43). Low-energy falls accounted for 65.1% (28/43) and higher-energy mechanisms 34.9% (15/43).

Fracture complexity was greater than in Cycle 1: complete-articular (AO C) fractures 69.8% (30/43), partial-articular (B) 23.2% (10/43), and extra-articular (A) 7.0% (3/43). This case mix reflects an appropriate selection of operative candidates.

Surgical Procedures Performed

ORIF with volar locking plate was undertaken in 40 patients (93.0%), all with intra-articular (B/C) fractures. Closed reduction with percutaneous K-wire fixation was used in three patients (7.0%), corresponding to extra-articular (A) patterns amenable to closed reduction. Technique selection was therefore compliant with NICE recommendation 1.4.6.

Compliance With Surgical Timing Guidelines

Overall compliance with NICE 1.4.3 timing standards was 11.6% (5/43). Table 1 summarises the findings.

**Table 1:** Compliance with NICE surgical timing standards NICE: National Institute for Health and Care Excellence

Fracture type	NICE standard	n (%)	Compliant n (%)	Mean time to surgery (days)	Range (days)
Intra-articular (AO B/C)	≤ 72 h (3 days)	40 (93.0%)	4 (10.0%)	8.6	2–21
Extra-articular (AO A)	≤ 7 days	3 (7.0%)	1 (33.3%)	6.3	4–9
Total	—	43 (100%)	5 (11.6%)	8.4	2–21

For intra-articular fractures, only 10% (4/40) met the ≤72-hour standard; the mean delay was 8.6 days. For extra-articular fractures, one-third (1/3) met the ≤7-day standard.

Distribution of Surgical Delays

Of 40 intra-articular cases: four (10%) within 72 hours (compliant), 12 (30%) within four to seven days, 18 (45%) within eight to 14 days, and six (15%) after > 14 days (maximum 21 days). Two extra-articular cases exceeded the seven-day limit (eight and nine days).

Barriers to Timely Surgery

The analysis of 38 non-compliant cases identified the following primary barriers in Table 2.

**Table 2:** Barriers to timely surgery The overwhelming contributor was restricted theatre access despite patients being fit for surgery.

Cause of delay	n (%) of delayed cases
Insufficient trauma-theatre capacity	28 (73.7%)
Medical optimisation (anticoagulation, cardiac review, diabetes control)	5 (13.2%)
Weekend / bank-holiday scheduling gaps	3 (7.9%)
Delayed inter-hospital transfer	2 (5.3%)

Comparison Between Audit Cycles

**Figure 1:**
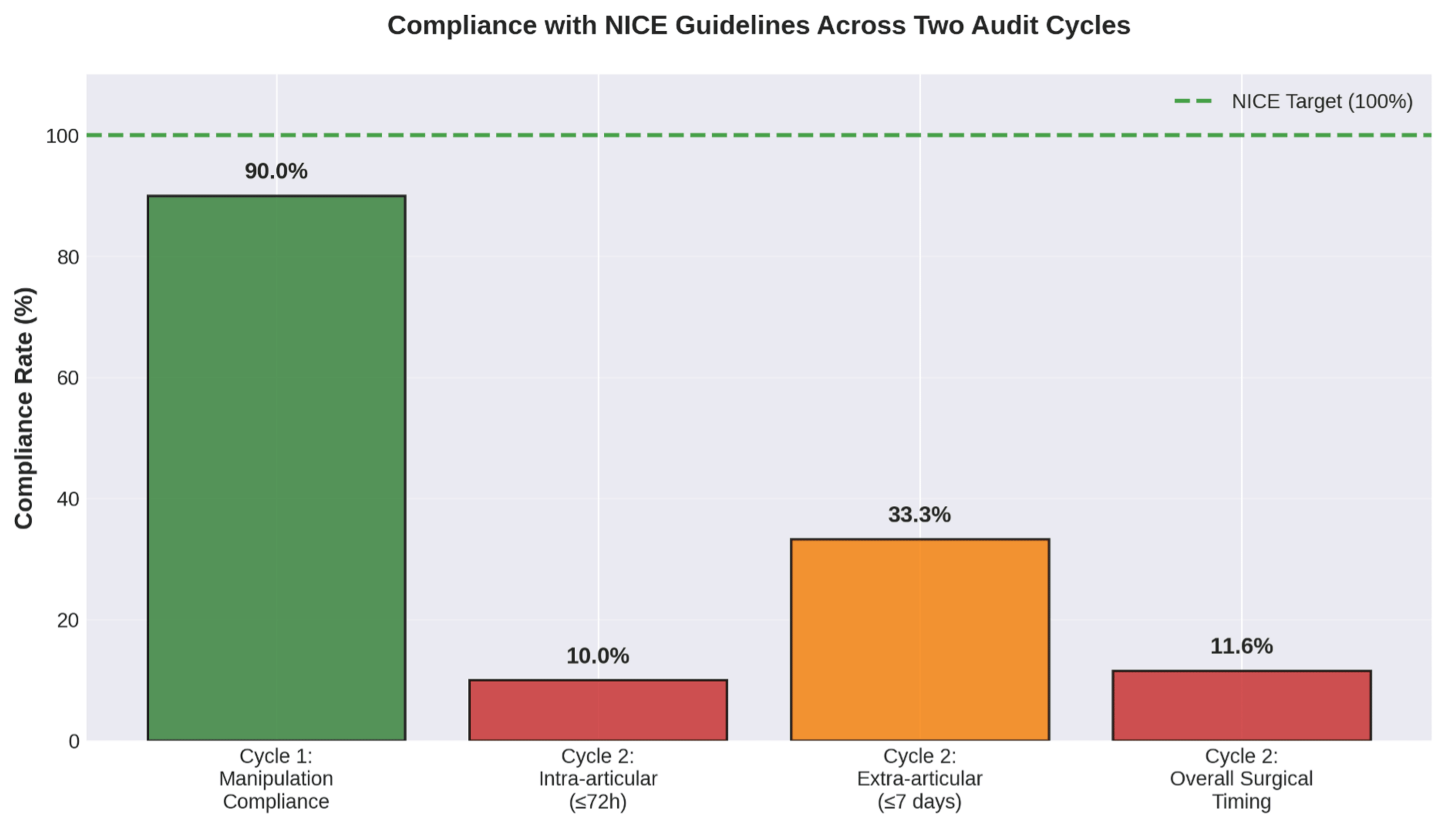
Comparison of compliance rates with the NICE guidelines across the two audit cycles The dramatic difference between cycles illustrates the distinction between knowledge-based barriers (successfully addressed through educational interventions) and system-level barriers (requiring organisational and resource allocation changes). NICE: National Institute for Health and Care Excellence This figure was created by the authors using Adobe Photoshop (Adobe Inc., California, USA).

Compliance improved for manipulation (90%) but remained poor for surgical timing (11.6%). Figure 1 illustrates the marked disparity, highlighting that educational interventions effectively corrected knowledge-based gaps, whereas theatre-capacity issues persisted as system-level barriers.

PDSA cycle summary

**Figure 2:**
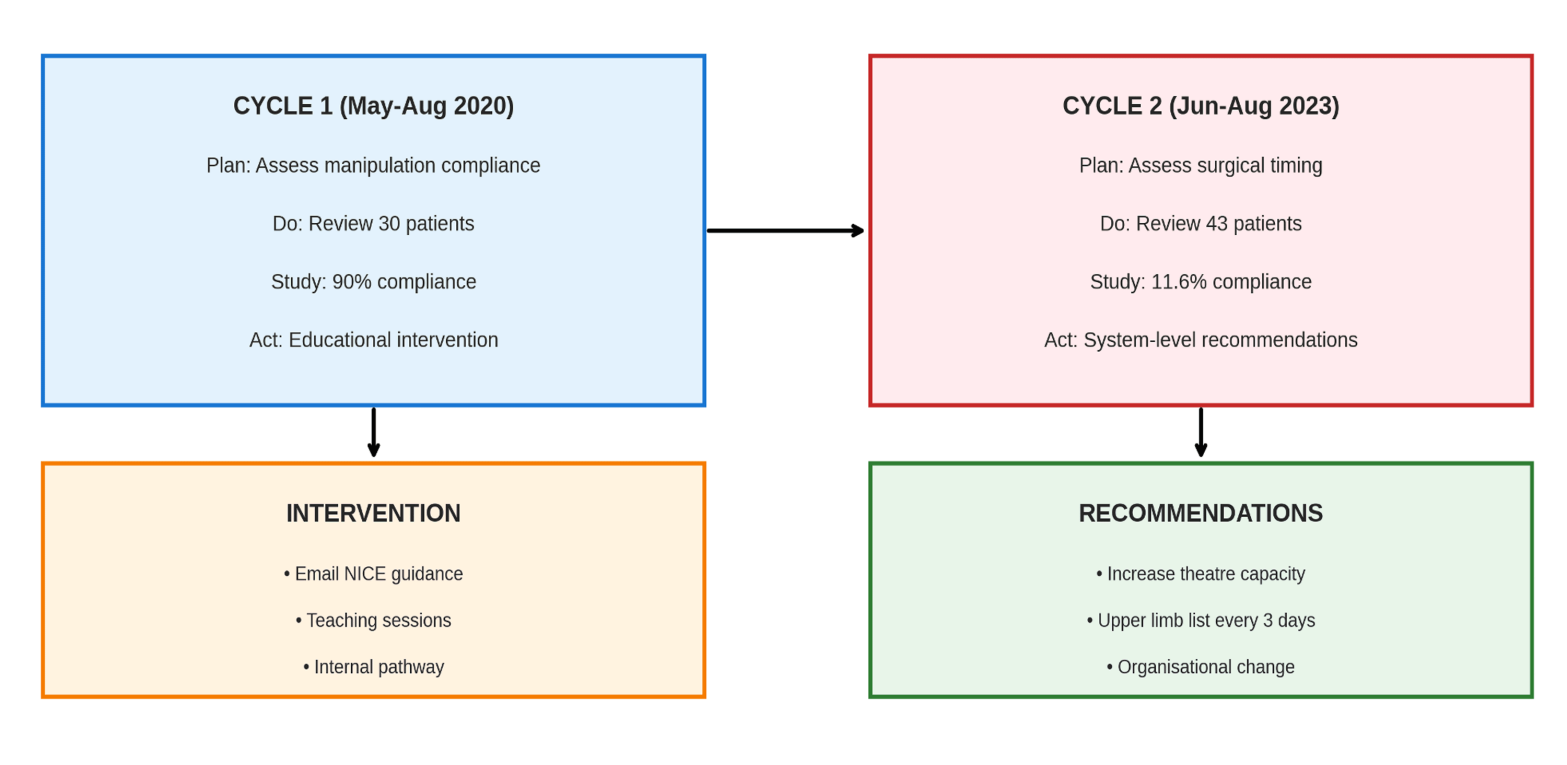
Plan-Do-Study-Act (PDSA) diagram demonstrating progression from baseline audit through educational intervention to re-audit and identification of system-level constraints. This figure was created by the authors using Adobe Photoshop (Adobe Inc., California, USA).

Figure 2 illustrates the complete PDSA process. Cycle 1 identified minor non-compliance in ED manipulation, prompting targeted education and guideline dissemination (Act Phase 1). Cycle 2 focused on surgical timing, revealing persistent organisational barriers despite good clinician knowledge. The next Act phase prioritises increasing trauma-theatre capacity and restructuring list allocation.

Summary of key findings

This two-cycle audit demonstrated that educational measures alone were effective in improving practitioner compliance but insufficient to overcome organisational bottlenecks. Cycle 1 achieved 90% compliance with manipulation standards, while Cycle 2 revealed severe under-performance in surgical timing (11.6%), driven chiefly by lack of theatre capacity (73.7% of delays). Mean time to surgery exceeded NICE targets by five to six days for intra-articular fractures and ~1 day for extra-articular fractures. Overall, clinicians applied appropriate surgical techniques consistent with NICE recommendations, indicating that further improvement depends on systemic resource and scheduling reforms rather than additional education.

## Discussion

This two-cycle quality improvement project highlights a fundamental difference between knowledge-based and system-level barriers to guideline compliance in the management of dorsally angulated distal radius fractures. However, the two audit cycles were conducted under different system conditions, with Cycle 1 taking place during the COVID-19 pandemic and Cycle 2 during the subsequent recovery phase. This reflects the real-world evolution of service pressures within the NHS and reinforces the central finding that structural capacity constraints, rather than knowledge deficits, remain the dominant barrier to timely surgery.

While the first audit cycle demonstrated strong baseline adherence (90%) to NICE guidance on manipulation, the second cycle revealed striking non-compliance (11.6%) with surgical timing standards. These contrasting results illustrate that educational interventions can improve awareness and documentation but are insufficient to overcome deeper organisational constraints.

Interpretation of findings

The high initial compliance with manipulation standards indicates that clinicians were already familiar with NICE recommendations and generally applied them appropriately. The small proportion of non-compliant cases in Cycle 1 largely reflected clinical exceptions, such as concomitant ipsilateral injuries or incomplete documentation, rather than true deviation from best practice. Educational sessions and targeted communication successfully reinforced this awareness.

By contrast, the poor adherence to surgical timing standards in Cycle 2 exposed a structural bottleneck rather than a knowledge deficit. Despite clinicians recognising the importance of timely fixation, most patients (88.4%) waited longer than the NICE-recommended intervals. This aligns with the CFIR, which identifies “inner-setting” factors, such as resources, staffing, and organisational culture, as critical determinants of implementation success [10]. The fact that nearly half of intra-articular fractures were operated on between eight and 14 days after injury, and 15% beyond two weeks, underscores that these were not minor scheduling lapses but systemic theatre-capacity limitations.

Comparison with existing literature

Our findings mirror wider evidence showing that failures in guideline implementation often arise from organisational barriers rather than individual clinician behaviour [8,9]. Fischer et al. found that workflow inefficiencies and resource scarcity are among the most common obstacles to evidence-based practice [8], while Wang et al. demonstrated that institutional infrastructure strongly predicts adherence to clinical guidelines [12]. Grol and Wensing further argue that educational strategies alone cannot address structural issues such as capacity or staffing [5,6].

Timeliness in fracture fixation has clear clinical relevance. Although data on distal radius fractures are limited, studies on hip and long-bone fractures consistently show that delayed surgery increases complication rates, hospital stay, and mortality [13]. NICE’s emphasis on early intervention therefore reflects a broader principle of trauma care, minimising pain, promoting early mobilisation, and optimising functional recovery.

Healthcare system implications

The audit’s findings have significant implications for resource planning. As the incidence of distal radius fractures rises with population ageing [3], orthopaedic departments face escalating demand for timely fracture care. Meeting NICE standards requires not only adequate theatre space but also coordinated scheduling, skilled multidisciplinary teams, and efficient patient throughput. Delays prolong hospitalisation, elevate complication risk, and inflate healthcare costs [2]. Yet, expanding theatre capacity demands considerable investment, forcing healthcare providers to weigh the clinical benefits of timely surgery against economic realities.

Barriers to implementation

The central barrier identified was inadequate theatre capacity to meet the operative demand within NICE timeframes. This constraint was multifactorial. Trauma theatres must accommodate a broad spectrum of urgent and emergency cases, and distal radius fractures, while important, are frequently deprioritised behind life- or limb-threatening injuries. Limited staffing, particularly shortages of anaesthetists, scrub nurses, and surgeons with upper-limb expertise, further restricted case throughput. Theatre scheduling was often inefficient, with the absence of dedicated upper-limb trauma lists forcing these cases to compete for general trauma slots. Predictable capacity shortfalls during weekends and public holidays also contributed to delays, particularly for patients injured late in the week. Together, these factors illustrate that improving compliance requires systemic change rather than isolated educational efforts.

Strengths and limitations

This project’s strengths include its two-cycle audit design, which enabled iterative learning, and its structured PDSA framework, ensuring systematic evaluation of interventions. The focus on nationally recognised NICE standards provided objective benchmarks for assessment. Comprehensive data collection across two cycles offered valuable insight into both clinical and organisational performance.

Nevertheless, limitations must be acknowledged. As a retrospective single-centre audit, the study relied on electronic records, which may have introduced documentation bias. The relatively small sample size, particularly for extra-articular fractures in Cycle 2, limits generalisability. Moreover, outcome measures such as functional recovery or patient satisfaction were not assessed, preventing evaluation of the direct clinical impact of delays. The three-year gap between cycles also introduces potential confounders such as staffing or service changes.

Recommendations for practice

Improving compliance with NICE standards demands a multifaceted organisational response. Expanding theatre capacity should be prioritised, whether through additional sessions or optimisation of existing ones. Establishing dedicated upper-limb trauma lists at regular intervals, ideally every three days, would provide predictable access for these patients. Developing a clear prioritisation protocol to ensure intra-articular fractures are operated on within 72 hours and extra-articular fractures within seven days would standardise decision-making. Enhanced communication between emergency, orthopaedic, and theatre teams, facilitated by daily trauma meetings, could streamline patient flow and reduce delays.

Maintaining surgical capacity during weekends and public holidays, either through on-call arrangements or extended weekday sessions, would further mitigate predictable scheduling gaps. Prospective monitoring of compliance via electronic dashboards could allow early detection of capacity issues and rapid intervention. Finally, engagement of hospital leadership and theatre management is essential to secure resources and institutional support. Demonstrating the patient-centred and economic advantages of guideline adherence may strengthen the case for investment.

Future directions

Sustained improvement will require commitment from multiple stakeholders. Future PDSA cycles should evaluate the impact of these organisational reforms on surgical timing and patient outcomes. Incorporating functional and economic outcome measures, such as length of stay, complication rates, and cost-effectiveness, will provide evidence for broader adoption. Sharing these findings across NHS trusts may facilitate collective learning, as many institutions face similar systemic pressures. Ultimately, national-level discussions on trauma service funding and theatre resource allocation may be necessary to ensure consistent adherence to evidence-based fracture-care standards.

## Conclusions

This two-cycle audit highlights a clear divide between knowledge-based and system-level barriers in managing dorsally angulated distal radius fractures. Educational interventions improved awareness and sustained high compliance with manipulation standards, but surgical timing remained poor due to limited theatre capacity. Achieving full NICE compliance will require organisational reform, expanding upper-limb theatre access, optimising scheduling, and improving multidisciplinary coordination. Sustainable improvement depends not only on clinician education but also on addressing the structural constraints that limit timely, evidence-based fracture care.
